# Malformation Pattern and Molecular Findings in the *FGFR1*-Related Hartsfield Syndrome Phenotype

**DOI:** 10.3390/medsci14010004

**Published:** 2025-12-22

**Authors:** Federica Gaudioso, Giulia Pascolini

**Affiliations:** 1 Radiodiagnostics Division, Bicocca University, 20126 Milan, Italy; f.gaudioso@campus.unimib.it; 2 Genetic Counseling Unit, Istituto Dermopatico dell’Immacolata, IDI-IRCCS, 00167 Rome, Italy

**Keywords:** split-hand/foot malformation (SHFM), *FGFR1*-related Hartsfield syndrome, *FGFR1*, *TP63*-associated ectodermal dysplasia

## Abstract

Background/Objectives: The Fibroblast Growth Factor Receptor 1 (*FGFR1*, MIM*136350) is a protein member of the fibroblast growth factor receptor (FGFR) family, with various biological functions, such as the normal development control. It contains an extracellular site for the ligand (three Ig-like domains, IgI, IgII, IgIII), a single transmembrane and a cytoplasmic protein tyrosine kinase (TK) domain. Variants in this gene have been associated with a wide spectrum of genetic disorders, including the clinical entity known as *FGFR1*-related Hartsfield or Hartsfield syndrome (HRTFDS, MIM#615465), which is an autosomal dominant or recessive disorder characterized by the clinical association of split-hand/foot malformation (SHFM) and holoprosencephaly (HPE). Dysmorphic facies, including cleft/lip palate, genitourinary anomalies, cardiovascular defects and intellectual disability/developmental delay (ID/DD) can also be a part of the clinical picture. Methods: The malformation phenotype of HRTFDS has been reviewed in 26 previously reported patients in terms of single congenital defects, mutational spectrum, impacted protein domains and inheritance. Molecular basis, clinical management, main differential diagnoses and genetic counseling were also illustrated. Results: SHFM was identified in every patient. The other main associated features included craniofacial defects, skeletal malformation identified at radiography, genitourinary anomalies, HPE and cardiovascular disorders. *FGFR1* causative variants mainly impact the TK domain and have a smaller impact on other protein sites (IgII, IgIII). Conclusions: This study extensively recapitulates the malformation phenotype associated with HRTFDS and the underlying molecular perturbations. A multidisciplinary clinical approach is fundamental, in which genetic counseling can have an important role. However, our results are partial and refer to a restricted number of patients, pointing out the necessity of other descriptions and similar research. Additional studies will expand clinical and molecular knowledge as well as further clarify the biological mechanisms.

## 1. Introduction

Pathogenetic heterozygous variants in the Fibroblast Growth Factor Receptor 1 gene (*FGFR1*, MIM*136350) have been associated with a wide group of genetic conditions, including hypogonadotropic hypogonadism 2 with or without anosmia (Kalmann syndrome, KAL2; MIM#147950) [1], trigonocephaly 1 (TRIGNO1, MIM#190440) [2], Osteoglophonic dysplasia (OGD; MIM#166250) [3], Pfeiffer (MIM#101600) [4] and Jackson-Weiss [5] (JWS; MIM#123150) syndromes. Thus, a broad phenotypic diversity is identifiable. A peculiar malformation complex consisting of HPE and SHFM is caused by anomalies in this gene [6]. This is a rare skeletal development disorder, namely *FGFR1*-related Hartsfield syndrome (HRTFDS, MIM#615465), which is characterized by a wide range of limb defects severity, comprising variable degrees of median clefts of the hands and/or feet (hypoplasia/absence of central rays), aplasia and syndactyly. Craniofacial dysmorphisms, including cleft lip/palate, genitourinary malformations, cardiovascular anomalies and intellectual disability represent other diagnosable clinical features.

To date, few patients with HRTFDS have been reported, and the entire phenotype is yet to be further delineated.

Here, a comprehensive and detailed review of the associated congenital defects is provided, also illustrating other important aspects regarding the disease.

The distinctive clinical presentation of the *FGFR1*-related Hartsfield syndrome is highlighted among genetic disorders with split of the extremities.

## 2. Relevant Sections

To identify previous articles describing patients with the *FGFR1*-related Hartsfield syndrome and a confirmed molecular diagnosis, we interrogated the online databases Pubmed (https://pubmed.ncbi.nlm.nih.gov) and EMBASE (www.embase.com).

**Figure 1:**
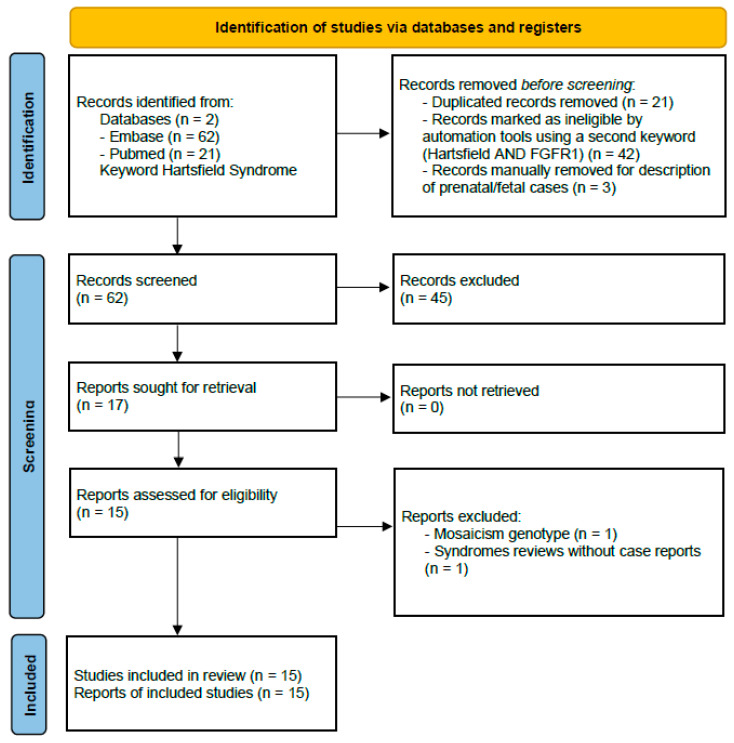
PRISMA diagram, showing the studies selection process of the present review.

Used research terms were *FGFR1* AND Hartsfield syndrome (HRTFDS) OR Hartsfield syndrome. Descriptions of prenatal cases or fetal presentation were not included. The review process is shown in the PRISMA diagram (Figure 1).

We identified 26 patients with a *FGFR1* pathogenic variant and a diagnosis of HRTFDS [6,7,8,9,10,11,12,13,14,15,16,17,18,19,20,21]. The research has been updated on 19 September 2025.

The main clinical findings are reported in Table 1. Causative variants, impacted protein domains, transmission model, gender distribution and clinical details for each patient are resumed in Supplementary Table S1.

Listed clinical findings are referred to the present review, which is devoted to the malformation spectrum in HRTFDS.

The SHFM and other rare associated malformations are illustrated as distinct paragraphs in the text.

### 2.1. Frequent Anomalies: The Split-Hand/Foot Malformation (SHFM) Phenotype

Abnormalities of the limbs were registered in a large part of patients (92%) and were represented by hand and feet split, often associated with syndactyly and oligodactyly. Two individuals were reported with isolated hand or feet oligodactyly/syndactyly; another patient presented with feet split and hand and feet syndactyly.

### 2.2. Other Associated Congenital Disorders

**Figure 2:**
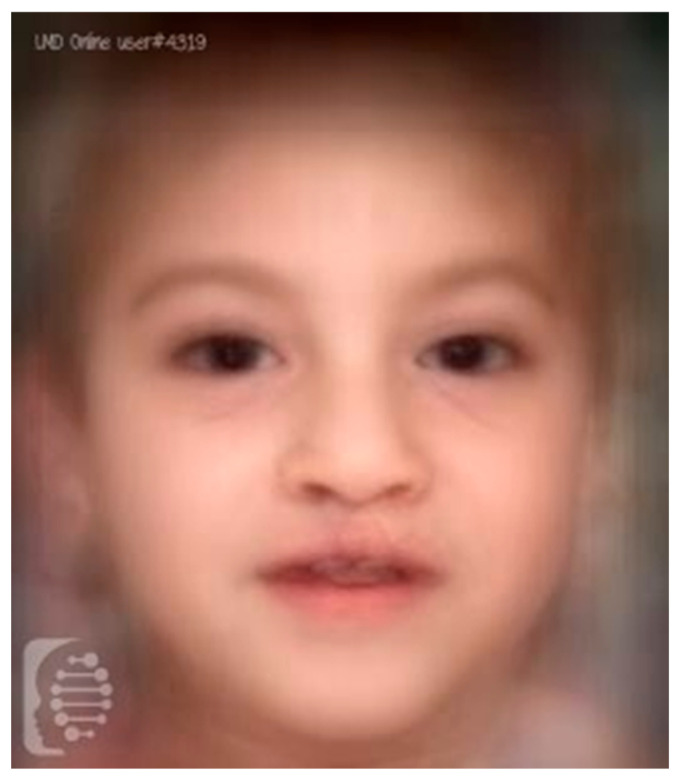
Facial mask/composite image of the craniofacial characteristics of the *FGRF1*-related Hartsfield syndrome. This composite image represents only a subset of facial features and may not capture the full phenotypic spectrum of HRTFDS. The image was generated and acquired from the archive of Face2Gene (F2G, www.face2gene.com, Boston, MA, USA). This technology uses a de-identified digital description of the face, starting from a clinical portrait. These composites are created by taking an average of the images that participated in the training of a syndrome in the system.

- Craniofacial anomalies were present in all patients (100%) and included cleft lip/palate, auricular malformations (outer ear anomalies, low set, microtia/small/cupped, dysplastic and skin tag), microcephaly, hyper/hypotelorism, nose anomalies (depressed nasal bridge, nasal tip clefting), midface and malar hypoplasia, retrognathia, abnormal oral cavity and telecanthus. One patient showed unilateral aural atresia and auricular pit, and other two individuals showed, respectively, (1) oligodontia of the permanent dentition, retention of multiple primary teeth, amelogenesis imperfecta and single central incisor with multiple hypodontia and (2) deformities of teeth 8 and 9, class II malocclusion, class II molar occlusion and palatal fistula, and anterior and inferior premaxilla were identifiable in an additional subject. Other singularly reported anomalies were high forehead, abnormal cranial sutures (prominent metopic and coronal sutures, wide patent sagittal and lambdoid sutures and anterior and posterior fontanelles), macrocephaly, colpocephaly, missing columella and glioma.
- Some of the main craniofacial features of HRTFDS are shown in Figure 2.
- Holoprosencephaly (lobar or semilobar) was identified in 90%, while corpus callosum (CC) anomalies, mainly consisting of partial or anterior portions or complete agenesis, were identified in 88%.
- Anomalies of the genitourinary tract, comprising cryptorchidism, hypoplastic testes and micropenis/microphallus were diagnosed in 88%.
- Cardiovascular malformations were recognizable in 80% and comprised aortic coarctation and isthmus stenosis. A single patient displayed lumbosacral spine vascular malformation associated with coarctation of the aorta.
- Skeletal anomalies and radiological findings were identified 100%. These principally included metacarpal or metatarsal fusions, rays’ absence, bones hypoplasia, dysplasia and syndactyly. Peculiar congenital malformations comprised thumbs bifurcation, metatarsal inverted Y synostosis and hand bifid distal phalanges; hip dysplasia was also reported.

### 2.3. Molecular Aspects

**Figure 3:**
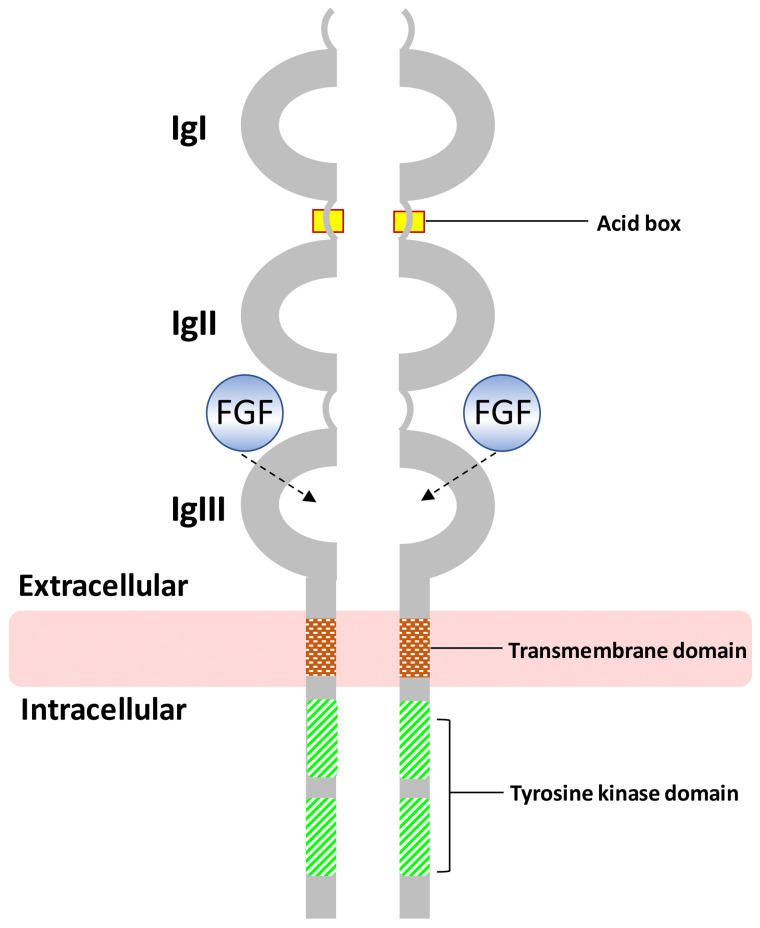
Schematic representation of the FGFR1 protein. The Immunoglobulin-like (IgI, IgII, IgIII), transmembrane and tyrosine kinase domains are shown. FGF bind site is also depicted. The figure is not drawn to scale and the depicted receptor consists of an FGFR1 protein homodimer.

The *FGFR1* gene, mapping on 8p11.23 chromosome region, plays a key role in mesoderm patterning, embryonic axial organization, skeletogenesis and development of the gonadotropin-releasing hormone (GnRH) neuronal system [22,23]. It comprises 24 exons, which are alternatively spliced, leading to different protein isoforms. The splicing process involves exons 8A or 8B, generating the two isoforms FGFR1-IIIb (also termed FGFR1b) and FGFR1-IIIc (also termed FGFR1c), respectively. These present different tissue distributions and FGF-binding affinities. The FGFR1-IIIc isoform has been found to be associated with most of the functions of *FGFR1*, contrary to FGFR1-IIIb, which seems to have a redundant functional role [24]. The full-length protein is a member of the Fibroblast Growth Factor receptors (FGFRs) family, which consists of an extracellular region (three immunoglobulin-like domains), a single hydrophobic membrane-spanning segment and a cytoplasmic tyrosine kinase domain (Figure 3) [25]. The amino acid sequence results are highly conserved between members and throughout evolution. The extracellular portion of the protein bounds the Fibroblast Growth Factor (FGF), promoting the dimerization of the receptor with any of the other four FGFRs (FGFR1-4) and the consequent cross-phosphorylation of the key tyrosine residues. This results in a setting in motion of a cascade of downstream cell signals, which depend on the cellular content in distinct cells and tissues. The main FGF/FGFR downstream signaling pathways comprise Ras/Raf-MEK-MAPKs (mitogen-activated protein kinases), phosphatidylinositol-3 kinase/protein kinase B (PI3K/AKT), PLCγ and the signal transducer and activator of transcription (STAT) [26].

**Figure 4:**
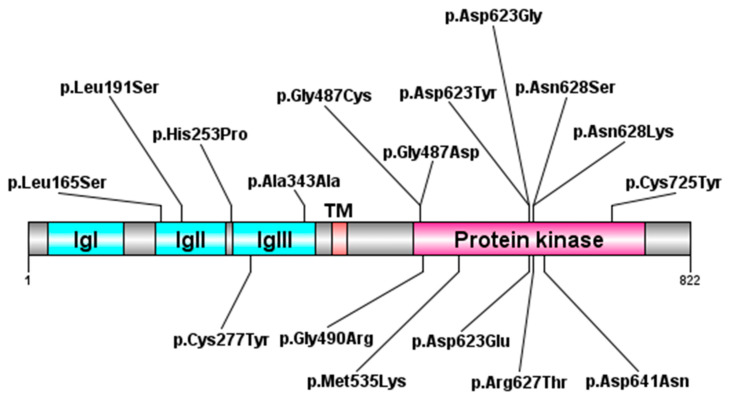
FGFR1 protein and disclosed *FGFR1* variants associated with the HRTFDS phenotype. The impacted domains are illustrated (IgI, Immunoglobulin-like domain I; IgII, Immunoglobulin-like domain II; IgIII, Immunoglobulin-like domain III, TM, transmembrane). Most variants fall within the protein kinase domain.

Based on the present review, *FGFR1* variants were missense and mainly located in the TK (tyrosine kinase) domain. The impacted domains were IgII and IgIII; no variants were found in the IgI and transmembrane (TM) domains (Supplementary Table S1 and Figure 4). The most recurrent nucleotide change was c.1880G>C(p.Arg627Thr), followed by c.1029G>A(p.Ala343Ala), c.1868A>G(p.Asp623Gly) and c.830G>A(p.Cys277Tyr) (Supplementary Table S1 and Figure 4).

### 2.4. Other Medical Issues and Global Clinical Management

Clinical and instrumental evaluation of the following clinical areas and associated disorders are recommended:

- Genetics, for clinical and molecular diagnosis, family and proband reproductive risks, coordination of the other medical specialists and cooperation with them.
- Neuropsychiatric, ID/DD, spasticity, seizures, temperature dysregulation, sleep–wake cycles disturbance, psychiatric conditions (anxiety, aggressive or self-injurious behavior) and feeding problems. Brain magnetic resonance imaging (MRI) can be used to investigate HPE and other CNS anomalies as well as electroencephalography (EEG) is suggested in case of seizures.
- Endocrine, for the central endocrine disorders, which are associated with midline brain defects, impairing the pituitary. These include growth hormone deficiency, central diabetes insipidus and hypogonadotropic hypogonadism.
- Craniofacial, for cleft lip/palate and related feeding problems.
- Musculoskeletal, for SHFM and motor impairment.
- Cardiovascular, for congenital heart and vascular diseases. Echocardiogram exam should be considered among diagnostic investigations.

Then, several specialistic medical figures are necessary to ensure correct and effective global management (clinical geneticist, adult/child neuropsychiatric, endocrinologist, orthopedic/rehabilitation therapist, cardiologist, maxillo-facial/plastic surgeon).

### 2.5. Malformation Phenotypes in Differential Diagnosis

The possible differential diagnoses of HRTFDS can include the other partially overlapping conditions, mainly manifesting with holoprosencephaly and SHFM.

- *TP63*-related disorders. This represents a wide group of genetic syndromes, which manifests with ectodermal dysplasia (hypohidrosis, nail dysplasia, sparse hair, tooth abnormalities), cleft lip/palate, SHFM/syndactyly. Other characteristics comprise lacrimal duct obstruction, hypopigmentation, hypoplastic breasts and/or nipples and hypospadias. The associated phenotypes are the Ectrodactyly, ectodermal dysplasia, cleft lip/palate syndrome 3 (EEC3; MIM#) [27], Acro-dermo-ungual-lacrimal-tooth (ADULT) syndrome (MIM#103285) [28], Limb-mammary syndrome (LMS, MIM#603543) [29] and Split-hand/foot malformation type 4 (SHFM4, MIM#605289) [30] and orofacial cleft 8 (OFC8, MIM#618149) [31]. The association of ankyloblepharon (tissue strands that completely or partially fuse the upper and lower eyelids), skin erosions especially on the scalp with areas of scarring, alopecia, trismus and excessive freckling describe the Ankyloblepharon-Ectodermal defects-Cleft lip/palate (AEC) syndrome or Hay–Wells syndrome (MIM#106260), comprising Rapp–Hodgkin syndrome (RHS, MIM#129400) [32].
- Genoa syndrome (MIM#601370), which is defined by semilobar holoprosencephaly, craniosynostosis and some skeletal anomalies such as small vertebral bodies, coxa valga and hypoplastic terminal phalanges of fingers [33]. Molecular bases are yet unknown.
- Duplication of the *ANOS1* gene (ANOSMIN 1, MIM*300836), associated with the X-linked Kallmann 1 syndrome (KAL1, MIM#308700), which can cause a phenotype reminiscent HRTFDS and consisting of hyperosmia, ectrodactyly, genital anomalies and mild ID [34].

### 2.6. Genetic Counseling and Inheritance

The *FGFR1*-related Hartsfield syndrome can be transmitted in an autosomal dominant or recessive manner.

- Autosomal dominant transmission.
  The condition is generally transmitted in an autosomal dominant fashion, and novel cases originate from a de novo mutational event, which can be confirmed by parental genetic testing. In the presence of a biologically confirmed paternal and maternal identity, the recurrence risk in these families is presumed to be low, and the chance to generate another individual with the condition depends on the presence of germinal (or somatic and germline) parental mosaicism, an event which has been previously described (Supplementary Table S1) [15,19,35].
- Autosomal recessive transmission.
  This model of inheritance has been observed (Supplementary Table S1) [6]. The reproductive risk will be 25% for two ascertained heterozygous carriers. If the pathogenic variant is identified in only one parent (and parental identity testing has confirmed biological maternity and paternity) and inherited by the son, the possible biological mechanisms leading to an affected individual could be (i) a de novo variant occurred in the wild-type allele in the index-case or (ii) a postzygotic de novo event in a mosaic parent [36]; (iii) uniparental isodisomy for the parental chromosome with the pathogenic variant could also be hypothesized.

## 3. Discussion

The *FGFR1*-related Hartsfield syndrome is a very rare genetic disorder with variably associated congenital defects. To date, known clinical data are related to a restricted number of patients, so this study contributes to the phenotypic recapitulation. In particular, the entire malformation pattern, molecular variants (with involved *FGFR1* protein domains) and transmission model have been reanalyzed in detail for each patient.

As can be extrapolated from the present study, split of the extremities in association with craniofacial dysmorphisms, genitourinary anomalies, HPE and CC defects represent recurrent features, followed by ear defects (which we considered as a clinical category separated from dysmorphia), labio/palatal and cardiovascular abnormalities (Supplementary Table S1). For patients with available details, the abnormal auricular morphology mainly consisted of microtia or short and low set/cupped ears (Supplementary Table S1).

HPE was principally lobar, while other recurrent CNS malformations resulted in abnormal olfactory bulbar, diminished cortical thickness and tethered spinal cord (Supplementary Table S1). At the radiological level, apart from all signs referring to split-hand/foot malformation, uncommon skeletal defects have been reported, such as thumbs bifurcation, metatarsal inverted Y synostosis and bifid distal phalanges of hand (Supplementary Table S1).

Concerning the single impacted FGFR1 domains, a mutational distribution could be disclosed, with most variants afflicting the TK domain (Supplementary Table S1 and Figure 4), suggesting that kinase-specific variants lead to the multisystem manifestations of HRTFDS, as previously demonstrated [16]. Specifically, *FGFR1* variants clustering in the TK domain underlie the disorder through a dominant-negative function, as demonstrated by in vivo models [16]. Conversely, gain and loss-of-function anomalies are considered responsible, respectively, for other allelic disorders, and specifically the *FGFR1*-related craniosynostosis/skeletal dysplasia and KAL2 [16]. Other damaging molecular mechanisms were previously explored. *FGFR1* perturbations in HRTFDS have been found to determine a dysregulated RAS/ERK1/2 pathway signaling, altering the autophagy process and the normal tissue homeostasis [17].

Differential diagnoses include some genetic conditions with multiple congenital anomalies. In particular, SHFM and clefting are important features also in the *TP63*-associated disorders, which represent a specific subset of ectodermal dysplasia. The recognition of this peculiar extremity’s defects can be considered a major criterion for the diagnosis, being identifiable in almost all these diseases. TP63 is a transcriptional factor whose anomalies have been demonstrated to alter limb, craniofacial and epithelial development in rats [37]. Interestingly, some skin and ectodermal characteristics have been observed in HRTFDS. In the present study, we identified one individual with sparse scalp, SPHFM, HPE and CC dysgenesis [6,10]. The underlying biological mechanisms possibly determining a skin/ectodermal phenotype in HRTFDS are not understood, and final conclusions cannot be drawn at this time. The hypothetical connections between *FGFR1* and *TP63* molecular pathways should then be clarified in the future.

A multidisciplinary clinical management is required, considering the polimalformation character of the syndrome. Then, the involvement of several medical professionals is necessary. Among them, geneticists can intervene in clinical and molecular diagnosis, offering genetic counseling to families. This can improve the knowledge of the syndrome and needs of patients as well as support in reproductive choices, including prenatal diagnosis and pre-implantation genetic testing.

### Limitations of the Present Review

The following limitations should be considered in this study:

- Small sample size effects on generalizability.
- Potential ascertainment bias in published case reports.
- Heterogeneity in clinical reporting across different centers.

## 4. Conclusions and Future Directions

This study represents a recent recapitulation of the ultrarare clinical entity termed *FGFR1*-related Hartsfield syndrome, which associates SHFM with craniofacial anomalies and HPE.

Data here presented are limited to a small sample of patients and certainly to be improved in the future. Some features may have been overlooked and not reported. Then, it is not possible to achieve robust statistical significance of the obtained results and definitive conclusions.

Additional descriptions are necessary to further delineate the clinical picture, expand the genotype–phenotype correlations as well as the knowledge about the underlying molecular pathways and their connections.

## Figures and Tables

**Table 1:** Main clinical features and frequencies of the *FGFR1*-related Hartsfield syndrome based on the present review. Frequencies are calculated as number of affected patients divided by total patients with available data for that feature.

System/Concern	Features	Frequencies (%)
Craniofacial	Abnormal outer ear	87%
	Oral cleft	76%
Limb	Radiologically identified skeletal defects	100%
	Split-hand/foot malformation	92%
Genitourinary	Penis/testes anomalies	100%
Central Nervous System (CNS)	Holoprosencephaly	90%
Cardiovascular	Aortic malformation	80%

## Data Availability

No new data were created or analyzed in this study.

## Supplementary Materials

The following supporting information can be downloaded at https://www.mdpi.com/article/10.3390/medsci14010004/s1, Supplementary Table S1: Malformation phenotype of the molecularly confirmed patients with the *FGFR1*-related Hartsfield syndrome phenotype.

## Abbreviations

The following abbreviations are used in this manuscript:

AEC	Ankyloblepharon-Ectodermal Defects-Cleft lip/palate
ADULT	Acro-dermo-ungual-lacrimal-tooth
ANOS1	Anosmin 1
CC	Corpus callosum
CNS	Central Nervous System
CL/P	Cleft lip/palate
DD	Developmental delay
EEC3	Ectrodactyly, ectodermal dysplasia, cleft lip/palate syndrome 3
FGF	Fibroblast Growth Factor
FGFR1	Fibroblast Growth Factor Receptor 1
FGFRs	Fibroblast Growth Factor Receptors
F2G	Face2Gene
HPE	Holoprosencephaly
HRTFDS	Hartsfield syndrome
H/F	Hands/feet
ID	Intellectual disability
IgI	Immunoglobulin-like I
IgII	Immunoglobulin-like II
IgIII	Immunoglobulin-like III
JWS	Jackson-Weiss syndrome
KAL1	X-linked Kallmann 1 syndrome
KAL2	Kalmann syndrome 2
LMS	Limb-mammary syndrome
TRIGNO1	Trigonocephaly 1
OGD	Osteoglophonic dysplasia
OFC8	Orofacial cleft 8
RHS	Rapp-Hodgkin syndrome
SHFM	Split-hand/foot malformation
SHFM4	Split-hand/foot malformation type 4
TP63	Tumor protein p63
TK	Tyrosine kinase
TM	Transmembrane
