# Supplementary material for: Malformation Pattern and Molecular Findings in the FGFR1-Related Hartsfield Syndrome Phenotype
Source: Med Sci (Basel). 2025 Dec 22;14(1):4. doi: 10.3390/medsci14010004 (PMC12821716; doi:10.3390/medsci14010004)
Supplement: Supplementary file 1 [file medsci-14-00004-s001.zip › medsci-3816260-supplementary.pdf]

Supplementary Table 1. Malformation phenotype of molecularly confirmed patients with the *FGFR1*-related Hartsfield syndrome phenotype. The Human Phenotype Ontology (HPO) number is indicated. The genotype (monoallelic or biallelic variant) is specified.

Reference	Patient (n.)	Genotype	Protein domain	Gender	Abnormal facial shap (HPO 0001999)	Abnormality of the skeletal system (radiological findings) (HPO 0000924)	Abnormality of the genitourinary system (HPO 0000119)	Abnormality of limbs (HPO 0040064)	Holoprosencephaly (HPO 0001360)	Other morphological CNS abnormality (HPO 0002011)	Abnormal corpus callosum morphology (HPO 0001273)	Abnormality of the outer ear (HPO 0000356)	Abnormality of the cardiovascular system (HPO 0001626)	Oral cleft (HPO 0000202)
Simonis et al.	1*	c.494T>C(p.Leu165Ser)/ c.494T>C(p.Leu165Ser)	IgII	M	+ (macrocephaly, prominent metopic and coronal sutures, widely patent sagittal and lambdoid sutures and anterior and posterior fontanelles, hypotelorism)	+ (two digits H, one digit F)	na	+ (split H/F)	+ (alobar)	+ (diminished cortical thickness)	+ (absent)	+	na	+ (large median CL/P)
	2	c.572T>C(p.Leu191Ser)/ c.572T>C(p.Leu191Ser)	IgII	M	+ (hypotelorism)	+ (left 6th metacarpal bones with 4th and 5th partial fusion)	na	+ (split H/F)	+ (lobar)	+ (diminished cortical thickness)	+	na	na	-
	3	c.1468G>C(p.Gly490Arg)	TK	M	+ (hypertelorism)	+ (hip dysplasia, thumbs biforcation, absent 2nd fingers, absent/dysplastic 3rd fingers, broad bases of 4th fingers)	+ (micropenis, cryptorchidism)	+ (split F, left camptodactyly H, 5th finger clinodactyly, equinovarus deformity)	+	na	na	na	na	+ (bilateral CL/P)
	4**	c.1867G>T(p.Asp623Tyr)	TK	F	na	+ (left foot: fusion of 1st and 2nd toes, large gap between 2nd and 3rd rays, syndactyly of toes 3–5, absence of the 3rd phalange of digits 3 and 4; right foot: central large gap with partial syndactyly of toes 3–5, absence of the 3rd phalange of digits 2 and 3)	na	+ (split F)	+ (lobar)	na	+ (partial hypoplasia)	na	na	-
	5***	c.1884T>G(p.Asn628Lys)	TK	M	+ (microcephaly, telecanthus, sparse scalp hair)	+ (absence of 2nd hands digits bilaterally, hypoplastic 3rd digit, fused 2nd and 3rd metacarpal bones)	+ (cryptorchidism, micropenis, small testes)	+ (split H/F)	+ (lobar)	+ (hyperintense signal of posterior pituitary)	+ (partial agenesis)	+	na	+ (bilateral CL/P)
	6****	c.2174G>A(p.Cys725Tyr)	TK	M	na	+ (3 digits of the left foot, 4 digits on the right)	+ (micropenis, bilateral orchidopexy)	+ (split H/F, syndactyly F)	+ (lobar)	na	+ (partial agenesis)	na	na	+ (large median CL/P)
Prasad et al.	1	c.1883A>G(p.Asn628Ser)	TK	M	na	+ (metatarsal inverted Y synostosis)	+ (microphallus)	+ (split H/F)	+ (semilobar)	na	na	+ (right sided microtia)	na	+ (bilateral CL/P)
Takagi et al.	1	c.758A>C(p.His253Pro)	IgIII	M	+ (depressed nasal bridge)	na	+ (micropenis, undescended testes)	+ (split H)	+ (semilobar)	+ (absence of the olfactory bulb and fusion of the olfactory gyri)	na	+	na	+ (CL/P)
Lansdon et al.	1	c.1459G>T(p.Gly487Cys)	TK	M	+ (deformities of teeth 8 and 9, class II malocclusion, class II molar occlusion, palatal fistula, anterior and inferior premaxilla)	+ (possible 3rd metacarpal without any phalanges bilaterally)	+ (small penis)	+ (split H/F)	na	+ (enlargement of the 3rd ventricle)	+ (agenesis)	+ (small, cup shaped, low set)	-	+ (bilateral CL/P)
Dhamija et al.	1*****	c.1880G>C(p.Arg627Thr)	TK	M	+ (microcephaly, midface hypoplasia, bilaterally low-set ears)	+ (absent central rays H/F)	+ (undescended testes, micropenis)	+ (split H/F)	+ (semilobar)	+ (midline fusion of caudate, small 3rd ventricle, mildly hypoplastic medial temporal lobes and hippocampi, tethered spinal cord)	+ (partial absence of the anterior of genu)	+	na	+ (bilateral CL/P)
	2	c.1880G>C(p.Arg627Thr)	TK	M	+ (microcephaly, midface hypoplasia)	na	+ (undescended testes)	+ (split H/F)	+ (semilobar)	+ (tethered spinal cord)	+	+	+ (aorta coarctation, lumbosacral spine vascular malformation)	+ (bilateral CL/P)

Oliver et al.	1	c.1880G>C(p.Arg627Thr)	TK	M	+ (microcephaly, hypertelorism, bilateral epicanthal folds, malar and midface hypoplasia, nasal tip clefting, retrognathia, single central incisor, malar hypoplasia)	na	na	+ (split H/F)	+	na	na	+ (low set ears, bilateral microtia, unilateral aural atresia, cupped-ear deformity, auricular skin tag, auricular pit)	na	+ (bilateral CL/P)
	2	c.1880G>C(p.Arg627Thr)	TK	M	+ (hypertelorism, midface and malar hypoplasia, nasal tip clefting, retrognathia)	na	+ (micropenis)	+ (split H/F)	+	na	na	+ (low set ears, cupped-ear deformity, auricolar skin tag)	+ (mild juxta ductal aortic coarctation)	+ (bilateral CL/P)
	3	c.1880G>C(p.Arg627Thr)	TK	M	+ (hypertelorism, midface and malar hypoplasia, bilateral epicanthal folds, nasal tip clefting and glioma, retrognathia)	na	na	+ (split H/F)	+ (semilobar)	na	na	+ (cupped-ear deformity)	+ (aortic coarctation)	+ (bilateral CL/P)
Hong et al.	2	c.1921G>A(p.Asp641Asn)	TK	F	+ (microcephaly)	na	na	+ (oligodactyly F, syndactyly H)	+ (semilobar)	+ (pituitary hypoplasia)	na	+ (dysplastic ears)	na	+ (bilateral CL/P)
	3	c.1880G>C(p.Arg627Thr)	TK	M	na	na	+ (undescended testes)	+ (split H/F)	na	na	+	+ (right microtia, left malformed ear)	na	+ (bilateral CL/P)
	4	c.1869C>G(p.Asp623Glu)	TK	na	na	na	na	+ (split H/F)	+	na	na	na	na	na
	5	c.1604T>A(p.Met535Lys)	TK	na	+ (single central incisor, missing columella, multiple hypodontia)	na	na	-	-	-	-	na	na	+ (bilateral CL/P)
	6	c.1460G>A(p.Gly487Asp)	TK	M	+ (high forehead, microcephaly, hypertelorism)	+ (absence of 2nd digit phalanges bilaterally F)	na	+ (split F; tapering of fingers, left 3rd digit, camptodactyly, bilateral 5th digit clinodactyly)	+ (lobar)	na	+ (absence of anterior part, absent settum pellucidum)	na	na	+ (bilateral CL/P)
Palumbo et al.	1	c.1883A>G(p.Asn628Ser)	TK	M	+ (oligodontia of the permanent dentition, retention of multiple primary teeth, amelogenesis imperfecta)	+ (bifid appearance of the distal phalanx of the left 3rd finger, metatarsal inverted Y synostosis)	+ (orchidopexy)	+ (split F, syndactyly foot)	+ (lobar)	na	+ (agenesis of the anterior part)	na	na	-
Courage et al.	1	c.1029G>A(p.Ala343Ala)	IgIII	M	+ (microcephaly, sagittal craniosynostosis, single maxillary incisor)	na	+ (cryptorchidism)	+ (split F; syndactyly H/F)	+ (lobar)	na	-	-	na	-
	2	c.1029G>A(p.Ala343Ala)	IgIII	F	+ (colpocephaly)	na	na	+ (polysyndactyly of right F)	-	na	+ (agenesis)	-	+ (aortic isthmus stenosis)	-
	3	c.1868A>G(p.Asp623Gly)	TK	M	+ (flat profile, arched eyebrows, thin vermilion border)	+ (abnormal metatarsals)	+ (micropenis, cryptorchidism, severe hypoplastic testes)	+ (split H/F)	+ (semilobar)	+ (absence of the cerebellar vermis)	+ (agenesis)	+ (low set and dysplastic ears)	na	+ (bilateral CL/P)
Kobayashi et al.	1	c.1868A >C(p.Asp623Gly)	TK	M	+ (midface hypoplasia)	+ (absent proximal phalanges H/F)	+ (micropenis, small testes)	+ (split H/F, syndactyly H)	+	+ (hypoplasia of the olfactory bulb)	+ (partial absence of anterior part)	na	na	+ (CL)
Szoszkiewicz et al.	1	c.830G>A(p.Cys277Tyr)	IgIII	M	na	+ (Cleft left foot and polydactyly of the third toe, first and second right toe syndactyly and polydactyly of third toe)	na	+ (split left F, synpolydactyly right F)	na	na	na	na	na	-
	2	c.830G>A(p.Cys277Tyr)	IgIII	M	+ (high forehead, narrow palpebral fissures, micrognathia, low set ears)	+ (bilateral anomalies of 2nd and 3rd metatarsals, irregular ossification centers of the hands)	na	-	na	na	na	na	na	+ (CL)
Tot.	26	Monoallelic 92% (n = 24); Biallelic 8% (n = 2)	TK 73% (n=19); IgIII 19% (n= 5);	M 89% (n= 21); F 11% (n= 3)	100% (n = 20)	100% (n = 15)	100% (n = 14)	92% (n =24)	90% (n = 20)	90% (n = 10)	88% (n = 14)	87% (n = 13)	80% (n = 4)	76% (n = 19)

IgII 8% (n=2)

Legend: *Vilain et al. 2009 patient 3; **Vilain et al. 2009 patient 5; ***Van Maldergem et al. 1992; Vilain et al. 2009 patient 2; Metwalley et al. 2012; ****Vilain et al., 2009 patient 4; *****Keaton et al. patient 13; ****** Takenouchi et al. 2012; IgII= immunoglobulin-like 2 domain; IgIII= immunoglobulin-like 3 domain; TK= tyrosine kinase domain; CNS= Central Nervous System; CL/P = cleft lip and/or palate; H/F = hands or feet; na = not available/not reported. n = total number of patients reported with that feature. "na" were not included in the final count. For the corresponding reference see the text.
